# Understanding Patient Perspectives on the Use of Gamification and Incentives in mHealth Apps to Improve Medication Adherence: Qualitative Study

**DOI:** 10.2196/50851

**Published:** 2024-05-14

**Authors:** Steven Tran, Lorraine Smith, Stephen Carter

**Affiliations:** 1 School of Pharmacy Faculty of Medicine and Health University of Sydney Camperdown Australia

**Keywords:** qualitative, patient, perspectives, gamification, incentives, mobile app, mobile health, mHealth, medication adherence, mobile phone

## Abstract

**Background:**

Medication nonadherence remains a significant health and economic burden in many high-income countries. Emerging smartphone interventions have started to use features such as gamification and financial incentives with varying degrees of effectiveness on medication adherence and health outcomes. A more consistent approach to applying these features, informed by patient perspectives, may result in more predictable and beneficial results from this type of intervention.

**Objective:**

This qualitative study aims to identify patient perspectives on the use of gamification and financial incentives in mobile health (mHealth) apps for medication adherence in Australian patients taking medication for chronic conditions.

**Methods:**

A total of 19 participants were included in iterative semistructured web-based focus groups conducted between May and December 2022. The facilitator used exploratory prompts relating to mHealth apps, gamification, and financial incentives, along with concepts raised from previous focus groups. Transcriptions were independently coded to develop a set of themes.

**Results:**

Three themes were identified: purpose-driven design, trust-based standards, and personal choice. All participants acknowledged gamification and financial incentives as potentially effective features in mHealth apps for medication adherence. However, they also indicated that the effectiveness heavily depended on implementation and execution. Major concerns relating to gamification and financial incentives were perceived trivialization and potential for medication abuse, respectively.

**Conclusions:**

The study’s findings provide a foundation for developers seeking to apply these novel features in an app intervention for a general cohort of patients. However, the study highlights the need for standards for mHealth apps for medication adherence, with particular attention to the use of gamification and financial incentives. Future research with patients and stakeholders across the mHealth app ecosystem should be explored to formalize and validate a set of standards or framework.

## Introduction

### Background

Medication adherence is defined by the World Health Organization as the extent to which a person’s behavior corresponds to their agreed health recommendations from a health care provider [1]. In high-income countries, such as Australia and the United States, the estimated average adherence rate has remained at approximately 50% over the last two decades [2]. Adherence rates have also been measured to be much lower in low-income countries [1]. The direct consequences of medication nonadherence are suboptimal clinical benefits leading to disease progression and complications, which in turn has an impact on patients’ quality of life. The impact of medication nonadherence on health is even more apparent in older or low-income people, with a substantial association between higher all-cause hospitalization and mortality [3,4].

Medication nonadherence also contributes to medication wastage and a substantial economic burden arising from the medical resources consumed to treat preventable health events, productivity loss, and loss of life. In Australia, medication nonadherence was estimated to have an economic burden of approximately Aus $10 billion (US $6.5 billion) annually in 2018 [5]. This economic burden is expected to be much higher since then, exacerbated by additional barriers to medication adherence, such as travel restrictions and medication shortages, from the recent COVID-19 pandemic [6] and financial stressors of a potential economic recession [7,8]. In light of the significant economic burden and health impact on patient lives, there is a pressing need to address medication nonadherence through health care–provided interventions [9].

More recently, mobile health (mHealth) apps have been used to support medication management and promote medication adherence [10]. Some mHealth apps use gamification to enhance user engagement and some also provide direct-to-patient financial incentives, which are funded by the government or third-party interest groups such as health insurers [11]. Gamification (ie, the use of game elements in nongame contexts) and financial incentives (ie, the provision of an item with real-world economic value in exchange for a completed task) have been applied across many health and nonhealth domains. Two notable examples of gamification and financial incentives are Duolingo (Duolingo, Inc) and the “Incentive to Quit” trial, respectively.

Duolingo is an educational technology company that has an app under the same name offering courses in multiple languages, music, and math. Currently, the app has over 21 million daily active users and has retained a high level of engagement over the last decade of operations attributed to its use of gamification such as point-based systems, social leaderboards, and interactive storytelling [12]. A meta-analysis on the gamification of learning [13] suggests that in addition to gamified storytelling, social interactions including competition and collaboration were particularly effective in facilitating behavioral and motivational learning outcomes. While it cannot be assumed that the competitive and collaborative aspects of gamification would foster behavioral or motivational outcomes for medication adherence, the meta-analysis [13] also notes that the self-determination theory by Ryan and Deci [14] can be used to explain the mechanistic effects of gamification in the context of learning. The self-determination theory has also been applied to health behavior change including medication adherence [15]. The theoretical framework outlines 3 psychological needs (ie, competence, autonomy, and relatedness) required for intrinsic motivation. This intrinsic motivation can subsequently result in volitional behavior such as improved medication adherence.

A Cochrane review [16] into incentives for smoking cessation found that not only the use of financial incentives, either monetary or vouchers, were accepted in multiple mixed-population settings but also there was sufficient evidence to indicate that incentives improved long-term smoking cessation rates. This outcome was also sustained after the withdrawal of the incentives. The result from the Cochrane review may have contributed to the inception of the “Incentive to Quit” trial, a government-funded program in Australia using supermarket vouchers for reaching smoking cessation milestones. The program will cost Aus $500,000 (US $324,255) with the aim to recoup the amount by reducing smoking-related public health costs that are estimated to be Aus $1.5 billion (US $972 million) nationally each year [17]. This signals a potential sustainable solution to funding concerns for financial incentive programs in countries providing universal health care.

In the domain of medication adherence, our scoping review [18] identified limited evidence for the benefit of gamification with incentives. We also highlighted a wide variation in app content, design, and development processes; the use of behavioral theories or frameworks; evaluation methods; and outcomes. The review also found that when there was no patient involvement, it was likely that design and implementation decisions were largely made by developers or researchers. This lack of patient involvement could lead to the misalignment of patients’ goals and bias in the selection and use of gamified and incentivized app features.

Several qualitative studies [19-21] have explored patient perspectives on the use of technologies such as mHealth apps for medication adherence. The studies include a range of findings including observed benefits of a single location to manage their whole regimen, the value of personalization and utility, and an ambivalent feeling toward apps in health. A limitation of these study findings is that they were specifically designed to investigate a particular chronic condition and may not apply to an app for a generalized cohort. Gamification and incentive-containing apps were also not the focus of those studies, limiting the ability to draw conclusions about how patients feel about apps using these features [19-21].

As there is insufficient knowledge in the existing literature pertaining to patient perspectives on gamification or financial incentives in a generalized cohort for medication adherence, a study was conducted to address this gap and disseminate the findings through publication.

### Objectives

This qualitative study aims to identify and understand patient perspectives of Australian patients taking medication for chronic conditions on the use of gamification and financial incentives in mHealth apps for medication adherence. The perspectives gained from this study will help identify barriers, identify potential opportunities, and provide a foundation for developers seeking to apply these novel features in an app intervention for a general cohort of patients.

## Methods

The COREQ (Consolidated Criteria for Reporting Qualitative Research) checklist was used to guide reporting [22]. A completed COREQ checklist applied to this study is available in Multimedia Appendix 1 [22].

### Ethical Considerations

Research involving humans in Australia is reviewed by an independent group of people called a Human Research Ethics Committee. The ethical aspects of this study have been approved by the Human Research Ethics Committee of the University of Sydney (project number 2022/061).

### Recruitment

Participants were passively recruited through study posters and flyers displayed by consenting pharmacies and medical practices and email newsletters distributed by patient advocacy groups (eg, organizations comprised of mostly patients or caregivers to represent and promote the needs and priorities of patients) to their members advertising the study. Participants who were interested self-enrolled via a QR code displayed on the recruitment materials, which directed them to a screening questionnaire. The screening questionnaire was based on the following inclusion criteria: aged ≥18 years, competent in English, taking ≥1 medication for an ongoing medical condition for ≥3 months, not being given end-of-life care or in palliative care, and not being a health care professional. Eligible participants were then contacted via phone by the first author (ST). The first author introduced himself by explaining his own professional background and personal motivations for conducting the study including that the research would contribute to a higher degree. The first author explained the purpose of the study and potential impact before confirming enrollment and availabilities for focus group sessions. Participants were assigned to groups based on their availability when at least 3 participants were available to attend a common session. An email was subsequently sent to the participant containing the link to the web-based focus group (Zoom; Zoom Video Communications Inc) and a link to a web-based survey (REDCap [Research Electronic Data Capture]; Vanderbilt University) to capture consent to record and the baseline characteristic questions.

### Focus Groups

In line with the COREQ checklist, the first author (ST), having worked in community pharmacies for 6 years, facilitated all the focus groups after undertaking formal training and orientation by the research team. Apart from the first author and participants, there were no other parties in any of the focus groups. All focus groups commenced with an introduction of the study topic and rules and guidelines for the focus group before the recordings were initiated. An ice breaker “1-fun fact” question was asked for all participants starting with the facilitator followed by a list of open-ended questions covering topics across medication adherence, gamification, and financial incentives. All focus groups were limited to a duration of 1 hour. Participants who attended the web-based focus group was compensated with an Aus $50 (US $32) e-gift card.

The semistructured focus group guide was created and reviewed by all authors to reduce assumptions and potential bias of the first author. The semistructured focus group guide is available in Multimedia Appendix 2. Using the constant comparative method, concepts raised in a focus group were used as additional prompts in the subsequent focus groups if it was not identified before moving on to the next topic. The additional prompts were neutrally phrased and open ended to limit bias. An example of an additional prompt is as follows: “In a previous focus group data management and privacy was mentioned, what are your thoughts on data management and privacy in an app like this?” The facilitator also actively aimed to explore positive and negative perspectives equally.

### Data Collection and Analysis

The web-based focus group sessions were audio and video recorded using Zoom and stored on a secured university-licensed cloud service (OneDrive; Microsoft Corp). Each recording was auto-transcribed (Adobe Premiere Pro; Adobe, Inc) before undergoing manual transcription by the first author for familiarization. Notes made by the first author during the focus groups were also annotated in the transcripts. A senior author (SC) reviewed the audio record and transcripts of the first 2 focus groups. Before conducting the remainder of the focus groups, the team discussed the conduct of the focus groups, discussed the preliminary findings, and provided advice to the first author regarding the use of prompts and pauses. The transcripts from 2 focus groups were independently reviewed and iteratively coded using NVivo (Release 1.7.1; Lumivero) into concepts by 2 of the authors (ST and SC). The research team then compared and discussed the concepts to generate a list of themes and subthemes. The subthemes and themes were evaluated and revised 3 times before being applied to the transcripts again for validation. Having decided on an agreed coding framework, this framework was applied to the remaining transcripts. Additional subthemes were added as required; however, the recruitment of the focus groups was discontinued when the last transcript did not generate any unique concepts indicating that the study was approaching data saturation. The last focus group, after recruitment ended, further indicated this as it also did not generate any unique concepts. The coding mapping tree is illustrated in Multimedia Appendix 3. Participants were informed of the preliminary findings, themes, and subthemes as a study summary and were invited to review the transcripts for commentary and correction. One participant concurred with our findings, and no other participant provided any feedback.

## Results

### Overview

Of the 20 participants who showed interest in participating in the study, 1 participant was excluded due to the contact being unreachable or unresponsiveness. A total of 19 participants were included in the 5 web-based focus groups (via Zoom) conducted between May 2022 and December 2022. The mean age of the participants was 40 (SD 17; range 19-71) years. All participants reported that they used their smartphone daily, while more than half reported playing games (13/19, 69%) and using loyalty rewards (12/19, 63%) on a weekly or daily basis. More than a third of the participants (7/19, 37%) were taking ≥3 medications. Further details on participant characteristics (Table 1) where collected and tabulated, such as self-reported clinical characteristics.

In total, 3 main themes were identified, along with 8 subthemes. Many of the concepts derived in the subthemes were interconnected and overlapped across the 3 main themes. Additional quotes from the participants corresponding to the subthemes are available in Multimedia Appendix 4.

**Table 1:** Demographic characteristics of the participants (N=19).

Characteristics		Values
Age (years), mean (SD; range)		40 (17; 19-71)
Sex (female), n (%)		12 (63)
**Frequency of phone use, n (%)**		
	Rare	0 (0)
	Weekly	0 (0)
	Daily	19 (100)
**Frequency of games played, n (%)**		
	Never	0 (0)
	Rarely	6 (32)
	Weekly	7 (37)
	Daily	6 (32)
**Frequency of loyalty rewards use, n (%)**		
	Never	0 (0)
	Rarely	7 (37)
	Weekly	9 (47)
	Daily	2 (16)
**Self-reported clinical characteristics, n (%)**		
	Arthritis and joint pain	4 (21)
	Cancer	1 (5)
	Cardiovascular disease	1 (5)
	Diabetes	3 (16)
	Kidney disease	2 (11)
	Mental and behavioral conditions	7 (37)
	Respiratory conditions	2 (11)
	Prefer not to disclose	2 (11)
	Other	7 (37)
**Number of medications, n (%)**		
	1	8 (42)
	2	4 (21)
	3	3 (16)
	4	1 (5)
	≥5	3 (16)
	Prefer not to disclose	0 (0)

### Theme 1: Novelty of Gamification and Incentives Require a Purpose-Driven Design

#### Overview

Game features designed to improve medication and health knowledge, the process of goal setting, and a sense of empowerment were recognized as potentially beneficial. The use of incentives arising from game features to drive medication adherence was a novel concept that was acceptable to some but not universally embraced. Given the novelty, participants expected apps with these features to have excellent functionality and reliability. They noted that potentially complex language, inaccessible terminology, and complicated medication use processes require that the digital usability of apps be optimized.

#### Gamification for Knowledge, Empowerment, and Goal Setting

Participants’ views about the use of gamification to drive medication adherence in apps were formulated from their knowledge and experience of using health apps in general. Participants’ expectations about apps for medication management centered on having reminders and features to help with the organization of supply. Some also had prior experience with medication apps that included resources to help them improve their medication knowledge. Those with no prior experience rationalized that this was a benefit for some but not necessarily for themselves. Participants believed that gamification could allow users to “test your knowledge before and after” use. One participant predicted that game features designed to educate about drug interactions could empower consumers to check, understand, and respond to drug interactions:

...to check the medications and the side effects and everything and whether if there’s any food or medicine interaction.

Participant 12

However, another participant doubted their capacity to benefit from this:

My chemist is frequently telling me don’t take this tablet with that tablet because they interact...If I had a more educational interactive session, I’m not sure the message might get through to an old head like this.

Participant 11

Participants expected game elements to ideally elicit and respond to the individuals’ specific health-related goals and personalized needs. In the following example, a participant shares how gamification had helped them to engage with yoga:

I think that gamification really was a selling point for me because to be honest. I...I wanted to be in the top batch (of users) and to do that I can’t be missing my exercise.

Participant 12

Failing to support or empower the user in response to individualized and time-sensitive goals would result in a lack of motivation to engage with gamification as expressed by Participant 2 in one focus group, which was endorsed by Participant 1 and 4:

I think when the goal goes away, I don’t feel the need to use the app anymore, I just stop using it completely.

Participant 2

The use of gamification to promote adherence seemed foreign to some; the following participant explained their concerns:

I have to say that it wouldn’t put me off, but I could see that some people would see it [gamification] as a trivialization of the process. And you know, on that, I don’t think it would encourage me either.

Participant 9

#### Incentives or Rewards for Driving Medication Adherence Is Novel

Most participants had experience with accruing and redeeming incentives in apps and understood that incentives create motivation to drive goals:

We are incentive driven as a race or as a people.

Participant 14

Participants expected incentivization to be facilitated by the accrual of points to culminate in a “tangible kind of incentive,” such as non–cash-purchasing power or other reward. There were mixed views about the notion of receiving monetary rewards for adherent behavior. Some embraced the notion, as can be seen in the following statement:

Something like that, that’s not cash or monetary appeals to me because it’s something I do to get a reward.

Participant 14

However, some explained that incentives were simply not needed:

And the only incentive for me is, it’s my health, it’s the motivating factor.

Participant 13

There was some innate hesitancy toward the concept of people receiving personal benefit for good medication adherence behavior:

I have a suspicion that I’m probably more adverse to having anything monetary related on apps, I think I suspect that I probably have a stronger aversion. So if you’re asking me if there was one, I probably would not use it. However, I don’t think the average population would have an issue if you combined financial incentive with nonfinancial incentive [features] or you could switch the feature on and off.

Participant 17

Some felt that simply rewarding the quantity of drugs dispensed could lead to perverse incentives:

...abuse of medications by people taking medications, you know, two or three times today when they're only prescribed to take it once a day to reach a financial incentive.

Participant 14

Furthermore, participants expressed the notion that receiving rewards for medication adherence could be problematic for people with gambling problems:

I guess one of my worries is that it can become a bit like gambling at some point, especially for people that are addiction prone, that’s the main thing that’s a worry for me when it comes to this sort of concept for medication adherence.

Participant 6

Regarding how the points were to be redeemed, some participants expressed that the accrual of points could be turned into financial rewards, redeemable in the pharmacy:

Maybe you get to 10% discount on your next to medication script.

Participant 12

Other participants wanted accrued points to turn into a notification that provides rewards at other vendors:

I’d much prefer to see a nonfinancial incentive something like your app flushes up a barcode for free coffee or a donut or whatever it is, you know, rather than a dollar value.

Participant 14

Some others wanted points to be converted to financial benefits for charitable organizations:

I would rather that I contributed to something that was part of a bigger pool of money to help people in need.

Participant 13

#### Functionality and Reliability

The participants also highlighted the importance of having a reliable and functional mHealth app to support medication adherence. This meant the need for an app to provide accurate information about their medication schedule and dosage, accurately keep track of their repeats, and keep reminding them when it was time to take their medication or when to fill their repeats. Apps that were slow and clunky also impacted their perception of its effectiveness to keep track of their medication. Overall, the participants were clear that a reliable and functional app was critical to their ability to successfully manage their medication adherence:

My scripts are different quantities, some of them fall due in like three weeks and some of them fall due in four weeks...the app wasn’t able to manage that [different frequencies of script repeat reminders], and it was easier for me to have the scripts (and manage it) myself.

Participant 14

The importance of reliability and consistency seemed particularly poignant when applied to the collation of rewards and incentives:

If there was like a problem with the app but their points whatever weren’t going through or they also had lots of the rewards got sold out and they couldn’t get what they wanted. I think those sorts of things could make people pretty unhappy and then they might just not use the app at all.

Participant 18

#### Digital Usability

It was considered imperative for an app to use simple, lay language to help break down complex medical topics or terminology. In addition, the app needs a simple user interface and intuitive features. Participants who identified as technology avoiders, due to a desire to be a digital minimalist or having concerns about their ability to use an app effectively, suggested that they would consider an app if the content was easy to understand and if it was not difficult to use. This can be further supported externally by a health care professional or care provider to guide the user or provide a tutorial or introduction to the app:

I've never used an app. I'm not very tech savvy. I'm probably tech phobic. I’m 60 and I’ve only had a mobile phone for the last two years... I would probably just need to someone to show me the benefits. How to make it [medication management] easy for me, how to use it [the app]. Probably they’re the main things for me.

Participant 13

Participants noted that digital usability can be enhanced using gamified graphical or visual representations to summarize complex topics and medication adherence numbers into easy-to-understand tallies and metrics:

...a good way to visualize a lot of like statistics and stuff rather than having it in words. And it’s all just this simple and easy to read, you know, you don’t have to have like a stats background.

Participant 19

### Theme 2: Trust-Based Standards

Participants expressed that their attitudes toward using gamification or financial incentives for medication adherence was highly influenced by their thoughts about whether the app was created and curated with trust-based standards. These standards related to the perceived credibility of the app and its ecosystem and the policies and governance relating to the user’s data.

#### Credibility of the App Ecosystem

An app ecosystem refers to the intricate network of connections among the app, devices, databases, and various stakeholders such as end users, developers, and app owners. Participants expressed that the credibility of an app’s ecosystem was determined by multiple factors such as brand image, mission statement, and history.

For example, some participants seemed reluctant or skeptical about the transfer of rewards being managed between commercial entities:

...the chemist to interact with the coffee vendor. And I can’t see that...business managing to cooperate appropriately.

Participant 11

Some expressed concerns that a transfer to charitable organizations is potentially problematic:

Really concerns me that the money wouldn't get to where it’s supposed to go, that you’re adding more and more middlemen to it and everybody takes their cut.

Participant 14

Credibility was noticeably different when financial incentives were provided by a for-profit versus a not-for-profit organization:

If it had some sort of backing from a site like GP’s or was from the government or from medical institutions or from medical groups, I would be more comfortable with it. And if it was just a private initiative, I wouldn't feel as comfortable around it, to be honest.

Participant 17

Generally, financial incentives provided by not-for-profit organizations were found to be more credible with most participants likely to engage with an app if it was managed by a not-for-profit organization. In contrast, participants were conflicted by for-profit organizations offering financial incentives as the app health benefits, enhanced by rewards, can be compromised and exploited. This was attributed to the lack of transparency over the economic sustainability of financial incentives. Credibility was also valuable in apps that did not offer financial incentives as it informed participants on the trustworthiness of educational medical content provided via gamified features.

#### Governance Over One’s Data

The notion of having to share one’s health data to engage with gamification and incentives was seen as highly sensitive. Most participants reported being somewhat comfortable with sharing some personal health data on the mHealth apps they had used. Moreover, when the practicalities of sharing private data to redeem rewards were discussed, participants expressed some skepticism:

When you try to fill in the forms [for financial incentives] and they ask you a lot of questions like your demographic, your age group and all. Even if they ask for my email and my name, that should be fine. But sometimes they’ve asked too many questions, and that won't appeal to me anymore because of data privacy concerns.

Participant 12

I would be averse to the model you proposed there. I don’t like the concept. It’s morally repugnant and that it comes to the measure of how much information do they wish me to provide at the point of redemption.

Participant 11

It was also noted that access to social game elements, including financial incentives or rewards, should not be dependent on the user’s decision to provide specific personal data. Having the ability for the app user to selectively choose what data are shared, including how far they are shared and for what purpose, was also suggested as a best practice by some participants. Participants wanted to be informed of what data would be shared before “consent to share” was requested.

### Theme 3: Personal Choice

The last theme was related to participants’ views about personal choice, that is, having the option to decide whether they should use an app and then how and when they would like to use the app with games and incentives.

#### Choice to Use the App

Many participants felt there was the potential for mHealth apps with gamification to effectively support medication adherence. Some would choose to engage with incentives. Participants also expected that medication management apps with gamification and incentives would come at no financial cost to them. Participants wanted the researchers to know that consumers did not want to feel forced or coerced into using these apps, especially by their treating health care provider or insurance provider:

I think it should be an option for everyone to use an app. So, whether they like it, I don’t think it should be forced upon anyone.

Participant 19

#### Ability to Customize and Choose What Features to Use in the App

There was significant variability among the participants on the extent to which they were required to engage with gamification and the accrual of incentives. It was evident that participants wanted the flexibility to decide how and when they wanted to use the app.

The participants noted that they wanted the option to toggle on and off features that they want to use or hide, respectively. An example of a specific feature customization is being able to change the esthetics and cosmetic features of the app to the user’s personal preference:

I mean you don’t want bright colors and childish kind of images for like a seventy-year-old. I feel like they want something more mellow and relaxed and I don’t know, I guess also like a customization of the actual theme. You know how sometimes like you can customize how you want something to look and make you feel that if you put it in the hands of the consumer, it can customize it. They’ll be more like your feel, more personal and they’ll enjoy using the app more.

Participant 19

While a high level of customizability was desired, it was acknowledged that this could increase complexity and potentially reduce digital usability. A balanced approach to customizability was suggested where the user would be provided with default “recommended” or standard features based on some characteristics of the user. For example, it was mentioned that it could be possible to provide a relatively younger person with a high degree of flexibility on first use. Whereas for an older person, the initial level of customization could be limited, while allowing options for further customization once the user was familiar with the basic functionality:

What puts me off is probably if it takes a long time to set up any customization or, you know, Avatar, that sort of stuff. And it took a long time for me, again, being very time poor, I think that would put me off. So maybe something very easy to navigate and stuff like that.

Participant 7

## Discussion

### Principal Findings

The themes synthesized in this study provide a preliminary understanding of patient perspectives on the use of gamification and financial incentives in mHealth apps to promote medication adherence. To summarize, participants expressed that apps that allow users access to game features, which are designed to promote knowledge about medicines, and allow the self-monitoring of medication taking were broadly accepted and even desirable. However, the notion that a user’s behavior would be monitored by a third party and that users may accrue financial or nonfinancial benefits as rewards for adherent behavior created some skepticism. An app would need to be underpinned by good governance that is built on trust in the sponsor for it to be adopted by the public. Participants believed that the adoption of apps with game features and incentives will require that users maintain a high level of personal choice in the selection of app features, desired engagement levels, and ways to redeem rewards.

In the focus groups, participants predicted that medication apps could be helpful for individuals taking multiple medications, for example, to help them overcome problems with forgetfulness or disarray, using reminders and scheduling. Participants in the focus groups tended to support the option of patients self-recording their medication consumption using a medication app. Previous research [15] suggests behavior change can be achieved with interventions based on the Self-Regulation Theory [23], for example, by optimizing attributes such as competence and autonomy. This could be explored through the self-monitoring of medication consumption using an app. With regard to gamification, participants saw potential to improve medication knowledge through engagement with gamified learning modules, which in turn could help patients to remain adherent to medicines for chronic diseases.

When the concept of potentially rewarding good medication adherence with financial or nonfinancial benefits was considered, participants discussed that to trigger and realize the incentives, their behavior would be observed by a third party that may or may not be their help providers. In research settings, the monitoring of adherence to provide incentives is achieved by directly observing consumption (ie, watching actual consumption in a community pharmacy), conducting pill counts of returned containers, and using electronic monitoring devices such as MEMS Caps. While those methods are accepted as accurate methods of measuring adherence [24], they are resource intensive, relatively intrusive, and unsuited for widespread adoption for the management of most chronic diseases. Therefore, while medications apps could be designed to monitor and incentivize adherence by communicating with an app, to the participants in this study, the notion of having their medication taking being monitored and receiving incentives for interacting with such an app was novel and generated several controversial discussions about appropriateness and ethicality of providing rewards for a health behavior. Much of the skepticism expressed was related to concerns about trust in third-party app providers.

The theme of trust-based standards aligns with existing and burgeoning concerns about data privacy and security across all mHealth apps; specifically with regard to inconsistencies in the way data privacy is applied [25]. In addition, Schroeder et al [26] found that the major concerns patients had on data privacy within mHealth apps were related to the potential risk of misuse of their personal health data and the fear of receiving personalized advertisements. In this study, participants actively vocalized a fear regarding which actors would have access to their health data and how private companies could monetize and gain from access to personal data. Commercial advertising and exploitation in an mHealth app setting raises substantial concerns on an ethical level, and revisions to consumer law are urgently needed to protect the user [27]. It is essential that apps adhere to established and emerging data privacy standards to establish a level of trust. While most health care apps do comply with the existing compliance standards [25,28], such as the General Data Protection Regulation and the Health Insurance Portability and Accountability Act, this is generally unbeknown to the public and considered inadequate by individuals when personally exposed to or made aware of events relating to security data breaches. This was particularly emphasized in our findings due to the recency of several major data breaches in Australia from a telecom company, Optus, and a health insurance company, Medibank, during the study period [29]. While this concern may have been inflated by recency bias, studies have reported increases in data breaches globally, indicating that these concerns may be warranted [30,31].

Further improvements in transparency and stronger preventive and remedial processes relating to data breach events or unauthorized access of data may alleviate this concern for the individual. Being data-privacy compliant and transparent adds to the security for an individual to carry out health activities within the app by mitigating the risk of damage to their identity and dignity [32]. When data privacy and security is applied and communicated effectively, the collection, analysis, use, and evaluation of personal health information have potential to generate new preventive and curative therapies, diagnostics, and the optimized delivery of health care. The well-managed use of app data when well managed can be fed back to the individuals as a form of digitally assisted precision medicine [33]. The concept of securely sharing data for the personal benefit of one’s medical treatment and no other uninformed purpose has been identified by our participants as an acceptable approach if it is provided as an opt-in option instead of a mandate of the app.

The likely success of mHealth apps with gamification and incentives largely depends on their ability to engage with their intended users and align with their needs and preferences. A SWOT (strengths, weaknesses, opportunities, and threats) analysis in the study by Hein et al [34] reported that while digital solutions add complexity to health regimens, they also have the potential to provide high-quality medication management, enable additional services, and drive innovation in health care. The added complexity of digital solutions can be contributed by potential issues with regard to functionality and reliability or poor digital usability. Ensuring the functionality, reliability, and digital usability of apps is paramount to all mHealth and IT solutions and is not exclusive to integrating gamification and financial incentives into mHealth apps for medication adherence. The findings of this study provide some anecdotal evidence of poor experiences with apps used in health and other areas. While general IT standards such as SQuaRE (System and Software Quality Requirements and Evaluation) exist, there are no gold standards to qualitatively evaluate software apps with regard to digital usability specifically for health [35-37]. The development of a framework to include and contextualize existing IT standards for health may provide further guidance when developing such interventions. This may include recommendations such as more rigorous testing, user feedback loops, and mechanisms to allow developers to identify and address any technical issues or usability challenges promptly. Furthermore, involving health care professionals and patients in the app’s design process can provide valuable insights into specific needs and preferences, ultimately enhancing engagement and adherence [38].

This study shows that potential users will want to maintain a high level of personal choice over how they opt in, configure, use, share their personal data, and opt out of mHealth apps with gamification and incentives. This finding highlights the critical role of ensuring that patients have a sense of autonomy over their health behavior [39]. As such, mHealth apps should be designed to empower patients to become active partners in their health care while ethically informing health care providers of patient-specific data. People who use mHealth apps have been shown to have increased satisfaction with their overall care and have reported improved interactions with their health care provider [40]. In addition, health care providers feel that the use of an mHealth app ensures better clinical decision-making and patient outcomes [41-43]. During clinical practice, the benefits of using an mHealth app should be outlined and promoted alongside their medical treatment. A holistic approach removes coercion and respects the patient’s autonomy over the app and their medical treatment. Sax et al [27] similarly argue that patients should have autonomy over their health care decisions and that more attention should be provided to mHealth apps due to the increasingly persuasive methods the apps use to influence the behavior of users potentially for economic gains. To analyze the ethical impact of an app on autonomy, Sax et al [27] further suggested a framework that considers 3 requirements: independence, authenticity, and options. Considering the role of the health care provider in mHealth, further research into best clinical practices on how to support mHealth adoption through these 3 requirements is needed.

In addition, personal choice is closely related to the self-determination theory by Deci and Ryan [23], which emphasizes the importance of autonomy, competence, and relatedness in motivation and behavior change. In the context of mHealth apps for medication adherence, this theory suggests that patients should be given a sense of control over their health care decisions and that the use of financial incentives and gamification should be aligned with their personal values and goals. From our scoping review [18], we analyzed various underpinning theories or frameworks used for app development and found that the self-determination theory was used among the included studies. This indicates that the self-determination theory could be a useful framework for bolstering an mHealth app designed to address medication adherence. Such an app might incorporate gamification and financial incentives, where participants noted that social features foster a sense of community, encouraging connection and relatedness. A further analysis of patient perspectives on the various gamified and incentivized features in the context of this framework may help generate the default “recommended” or standard features of an app. Research into best practices specifically for financial incentives in medication adherence would also help address the concerns of exploitation and abuse, which have been identified in our findings and the literature, to undermine intrinsic motivation [44].

Government agencies are making efforts to manage the challenges and facilitate the opportunities associated with mHealth apps in general, which will be important to the application of gamification and financial incentives to promote medication adherence. For example, during the study and analysis, the Australian Digital Health Agency published an assessment framework for mHealth apps [45] in December 2022. The Australian Digital Health Agency is a statutory agency of the Australian government aimed at accelerating the adoption and innovation of digital technologies for health. The framework is intended to be used as a reference tool for app developers working on health apps in Australia. While not specific to medication adherence, gamification, or financial incentives, we found that there were similarities between the derived themes and subthemes in this study and the assessment domains (namely acceptability, safety and trust, ease of use, privacy and security, and technical quality assurance).

### Contribution to Research

Overall, this study builds upon existing literature across medical modalities and conditions in mHealth apps to explore consumer perceptions of gamification or financial incentives for medication adherence. While some consumers appear ready to embrace the concept, it was surprising that participants ascribed such high importance to the notion that the widespread uptake of these features will require excellent governance and oversight. The themes have practical implications as a foundation for providing guidance to aspiring app developers. Further consultation with consumers, the industry, the government, and health providers will be required to ensure that apps using gamification and financial incentives are created and curated with clear guidelines and standards. It is also recommended that as the industry adopts these standards to create a specific app or app features, consumers are consulted throughout the design process, from conceptualization through early adoption and delivery to quality assurance. Future research is still warranted to discuss and evaluate the implementation of standards or frameworks in app development such as that provided by the Australian Digital Health Agency by consulting with more patients and industry stakeholders. This will ensure that the apps are acceptable and remain relevant and motivational to improve uptake.

### Limitations

Two main limitations were identified in the design and analysis of this study that may have affected the interpretation of reported results. The first limitation source comes from the recruitment strategy. The need to scan a QR code to self-register interest in the study may have excluded certain participant groups such as those who identify as digital minimalists or technology adverse despite meeting the inclusion criteria. While the QR code ensures we recruited appropriately skilled participants to the study, there is a risk of unintentionally excluding certain participants resulting in the loss of some opinions on this topic. In addition, our recruitment strategy was not specifically designed to support a demographically diverse set of participants, with regard to language or the cultural background. The small sample size may have further impacted the lack of diversity. The use of focus groups, while benefiting from fruitful discussions among participants, also inherently includes limitations in the form of conformity bias as there may be disproportionate contributions by the participants and heterogeneity in character personalities.

The second limitation arose because the facilitator, analysts, and authors were from health care backgrounds, which potentially introduces bias in study findings through moderating the focus groups and analysis of the data. While all authors were also health care consumers, future studies could include a stronger focus on personal reflexivity and involve a non–health professional and consumer in the research team.

Ultimately, while recruitment stopped because of signals indicating data saturation, the findings of this study are not intended to be taken as a final, comprehensive, and conclusive report on patient perspectives. As technology advances and changes, so do the public and individual perceptions of it. Since this study did not present participants with a working or beta model of a particular app, the findings are mainly limited to perspectives of the concepts of gamification and incentives in general, rather than any specific app.

### Conclusions

Our findings provide an introductory understanding of patient perspectives on mHealth apps including gamification or financial incentives for medication adherence. Developers seeking to apply gamification and incentives in a general cohort of patients should strive to involve patients and their perspectives in all stages to inform design and development. This is critical, given the variation in consumer attitudes to the way incentives could be operationalized and concerns about sharing their personal data.

In addition, trust-based standards and personal choice and autonomy should be respected to support optimal app development in this modality. These considerations can effectively be summarized by using a comprehensive and validated framework. This field would gain from further research in the discussion and evaluation of such frameworks and share best practices with further patients and industry stakeholders.

## Appendix

Multimedia Appendix 1 COREQ (Consolidated Criteria for Reporting Qualitative Research) checklist applied to “understanding patient perspectives on the use of gamification and incentives in mobile health applications to improve medication adherence.”.

Multimedia Appendix 2 Semistructured focus group guide.

Multimedia Appendix 3 Coding mapping tree.

Multimedia Appendix 4 Additional quotes from participants.

## Abbreviations

COREQ: Consolidated Criteria for Reporting Qualitative Research
mHealth: mobile health
REDCap: Research Electronic Data Capture
SQuaRE: System and Software Quality Requirements and Evaluation
SWOT: strengths, weaknesses, opportunities, and threats
