# Supplementary material for: Understanding Patient Perspectives on the Use of Gamification and Incentives in mHealth Apps to Improve Medication Adherence: Qualitative Study
Source: JMIR Mhealth Uhealth. 2024 May 14;12:e50851. doi: 10.2196/50851 (PMC11134245; doi:10.2196/50851)
Supplement: Multimedia Appendix 1 [file mhealth_v12i1e50851_app1.docx]

Multimedia Appendix 1: COREQ (COnsolidated criteria for REporting Qualitative research) Checklist applied to “Understanding Patient Perspectives on the use of gamification and incentives in mobile health applications to improve medication adherence”

| **Topic** | | **Item No.** | | **Guide Questions/Description** | **Reported in section** |  |
| --- | --- | --- | --- | --- | --- | --- |
| **Domain 1: Research team**  **and reﬂexivity** | | | | | |  |
| *Personal characteristics* | | | | | |  |
| Interviewer/facilitator | | 1 | | Which author/s conducted the interview or focus group? | Methods: Qualitative Focus Groups (*In line with the COREQ checklist, the first author (ST), having worked in community pharmacies (6 years) facilitated all the focus groups …*) |  |
| Credentials | | 2 | | What were the researcher’s credentials? E.g. PhD, MD | Declaration section/Author contributions (*ST, male industry professional (BPharm) – designed the study, collected data, and undertook coding and analysis and drafting of the manuscript. SC, male senior lecturer (BPharm, MSc, PhD, Grad Cert Edu (Higher Ed), FPS, FHEA) – designed the study, undertook coding and analysis and review of the manuscript. LS, female professor (BA (Hons)(Psych), PhD) – designed the study, adjudicated the coding and analysis and review of the manuscript.)* |  |
| Occupation | | 3 | | What was their occupation at the time of the study? |  |  |
| Gender | | 4 | | Was the researcher male or female? |  |  |
| Experience and training | | 5 | | What experience or training did the researcher have? | Methods: Qualitative Focus Groups (*In line with the COREQ checklist, the first author (ST), having worked in community pharmacies (6 years) facilitated all the focus groups after undertaking formal training and orientation by the research team.*) |  |
| *Relationship with*  *participants* | | | | | |  |
| Relationship established | | 6 | | Was a relationship established prior to study commencement? | Declaration section/Author contributions (*The participants did not have a professional or personal relationship with the researchers prior to the study.*) |  |
| Participant knowledge of  the interviewer | | 7 | | What did the participants know about the researcher? e.g. personal goals, reasons for doing the research | Methods: Recruitment (*Eligible participants were then contacted via phone by the first author (ST). The first author introduced himself by explaining his own professional background and personal motivations for conducting the study including that the research would contribute to a higher degree. The first author explained the purpose of the study and potential impact before confirming enrolment and availabilities for focus group sessions.)*  Methods: Qualitative Focus Groups (*An ice breaker “1-fun fact” question was asked for all participants starting with the facilitator …*) |  |
| Interviewer characteristics | | 8 | | What characteristics were reported about the inter viewer/facilitator? e.g. Bias, assumptions, reasons and interests in the research topic | Methods: Recruitment (*personal motivations for conducting the study including that the research would contribute to a higher degree)*  Methods: Qualitative Focus Groups (*The semi-structured focus group guide was created and reviewed by all authors to reduce assumptions and potential bias of the first author.*)  Methods: Qualitative Focus Groups *(The additional prompts were neutrally phrased and open-ended to limit bias. An example of an additional prompt was: In a previous focus group data management and privacy was mentioned, what are your thoughts on data management and privacy in an app like this? The facilitator also actively aimed to explored positive and negative perspectives equally.)* |  |
| **Domain 2: Study design** | | | | | |  |
| *Theoretical framework* | | | | | |  |
| Methodological orientation and Theory | | 9 | | What methodological orientation was stated to underpin the study? e.g. grounded theory, discourse analysis, ethnography, phenomenology,  content analysis | Abstract (*Transcriptions were independently coded to develop a set of themes.*)  Methods: Data collection and analysis section describes the methodological orientation in further details i.e., Thematic analysis. |  |
| *Participant selection* | | | | | |  |
| Sampling | | 10 | | How were participants selected? e.g. purposive, convenience,  consecutive, snowball | Voluntary sampling  Methods: Recruitment (*Participants were passively recruited through study posters and flyers displayed by consenting pharmacies and medical practices, and email newsletters distributed by patient advocacy groups (organizations comprised of mainly patients or caregivers to represent and promote the needs and priorities of patients) to their members advertising the study. Participants who were interested self-enrolled via a QR code displayed on the recruitment materials which directed them to a screening questionnaire.*) |  |
| Method of approach | | 11 | | How were participants approached? e.g. face-to-face, telephone, mail,  email |  |  |
| Sample size | | 12 | | How many participants were in the study? | Results (*A total of 19 participants were included in the 5 online focus groups (via Zoom) conducted between May 2022 and December 2022.*) |  |
| Non-participation | | 13 | | How many people refused to participate or dropped out? Reasons? | Results (*Of the twenty participants who registered interest in participating in the study, one participant was excluded due to contact unreachable/unresponsiveness.*) |  |
| *Setting* | | | | | |  |
| Setting of data collection | | 14 | | Where was the data collected? e.g. home, clinic, workplace | Methods: Data collection and analysis (*The online focus group sessions were audio and video recorded using Zoom and stored on a secured university-licensed cloud service (OneDrive). Each recording was auto-transcribed (Adobe Premiere Pro) before undergoing manual transcription by the first author for familiarization. Notes made by the first author during the focus groups were also annotated in the transcripts.*) |  |
| Presence of non-  participants | | 15 | | Was anyone else present besides the participants and researchers? | Methods: Qualitative Focus Groups (*Apart from the first author and participants, there were no other parties in any of the focus groups.*) |  |
| Description of sample | | 16 | | What are the important characteristics of the sample? e.g. demographic  data, date | Results (*A total of 19 participants were included in the 5 online focus groups (via Zoom) conducted between May 2022 and December 2022. The mean age of the participants were 40 years old (range 19-71). All participants reported that they used their smart phone daily, whilst more than half reported playing games (13/19, 69%) and using loyalty rewards (12/19, 63%) on a weekly or daily basis. More than a third of the participants (7/19, 37%) were taking 3 or more medications. Further details on participant characteristics (Figure 1) where collected and tabulated such as self-reported clinical characteristics.*) |  |
| *Data collection* | | | | | |  |
| Interview guide | | 17 | | Were questions, prompts, guides provided by the authors? Was it pilot  tested? | Methods: Qualitative Focus Groups (*The semi-structured focus group guide was created and reviewed by all authors to reduce assumptions and potential bias of the first author. The semi-structured focus group guide is available in Multimedia Appendix 2.*) |  |
| Repeat interviews | | 18 | | Were repeat inter views carried out? If yes, how many? | N/A, no repeat interviews were conducted with the same participants. |  |
| Audio/visual recording | | 19 | | Did the research use audio or visual recording to collect the data? | Methods: Data collection and analysis (*The online focus group sessions were audio and video recorded using Zoom and stored on a secured university-licensed cloud service (OneDrive).*) |  |
| Field notes | | 20 | | Were ﬁeld notes made during and/or after the interview or focus group? | Methods: Data collection and analysis (*Notes made by the first author during the focus groups were also annotated in the transcripts.*) |  |
| Duration | | 21 | | What was the duration of the inter views or focus group? | Methods: Qualitative Focus Group (*All focus groups were limited to a duration of one hour.*) |  |
| Data saturation | | 22 | | Was data saturation discussed? | Methods: Data collection and analysis (*Additional sub-themes were added as required; however, the recruitment of the focus groups was discontinued when the last transcript did not generate any unique concepts indicating that the study was approaching data saturation. The last focus group, after recruitment ended, further indicated this as it also did not generate any unique concepts.*) |  |
| Transcripts returned | | 23 | | Were transcripts returned to participants for comment and/or correction? | Methods: Data collection and analysis (*Participants were informed of the preliminary findings, themes and sub-themes as a study summary and were invited to review the transcripts for commentary and correction. One participant concurred with our findings and no other participant provided any feedback.*) |  |
| **Domain 3: analysis and**  **ﬁndings** | | | | | | |
| *Data analysis* | | | | | | |
| Number of data coders | | 24 | | How many data coders coded the data? | | Methods: Data collection and analysis (*The transcripts from two focus groups were independently reviewed and iteratively coded using NVivo (Release 1.7.1) into concepts by two of the authors (ST & SC).*) |
| Description of the coding  tree | | 25 | | Did authors provide a description of the coding tree? | | Methods: Data collection and analysis (*The research team then compared and discussed the concepts to generate a list of themes and sub-themes. The sub-themes and themes were evaluated and revised three times before being applied to the transcripts again for validation. Having decided on an agreed coding framework, this framework was applied to the remaining transcripts. Additional sub-themes were added as required... The coding mapping tree is illustrated in Multimedia Appendix 3.*) |
| Derivation of themes | | 26 | | Were themes identiﬁed in advance or derived from the data? | | Themes derived from the data.  Methods: Data collection and analysis (*The research team then compared and discussed the concepts to generate a list of themes and sub-themes. The sub-themes and themes were evaluated and revised three times before being applied to the transcripts again for validation.*) |
| Software | | 27 | | What software, if applicable, was used to manage the data? | | Methods: Data collection and analysis (*The transcripts from two focus groups were independently reviewed and iteratively coded using NVivo (Release 1.7.1) into concepts by two of the authors (ST & SC).*) |
| Participant checking | | 28 | | Did participants provide feedback on the ﬁndings? | | Methods: Data collection and analysis (*Participants were informed of the preliminary findings, themes and sub-themes as a study summary and were invited to review the transcripts for commentary and correction. One participant concurred with our findings and no other participant provided any feedback.*) |
| *Reporting* | | | | | | |
| Quotations presented | | 29 | | Were participant quotations presented to illustrate the themes/ﬁndings?  Was each quotation identiﬁed? e.g. participant number | | Yes, Results section provides illustrative quotes for each sub-theme with each quotation identified with the participant number. |
| Data and ﬁndings consistent | | 30 | | Was there consistency between the data presented and the ﬁndings? | | Results & Discussion section outlines consistency between data and findings. |
| Clarity of major themes | | 31 | | Were major themes clearly presented in the ﬁndings? | | Yes, Results section provides a descriptive explanation for each theme. |
| Clarity of minor themes | | 32 | | Is there a description of diverse cases or discussion of minor themes? | | Yes, Results section provides a descriptive explanation and illustrative quote for each theme and sub-theme. |

Reference: Tong A, Sainsbury P, Craig J. Consolidated criteria for reporting qualitative research (COREQ): a 32-item checklist for interviews and focus groups. *International Journal for Quality in Health Care*. 2007. Volume 19, Number 6: pp. 349 – 357
