# Supplementary material for: Understanding Patient Perspectives on the Use of Gamification and Incentives in mHealth Apps to Improve Medication Adherence: Qualitative Study
Source: JMIR Mhealth Uhealth. 2024 May 14;12:e50851. doi: 10.2196/50851 (PMC11134245; doi:10.2196/50851)
Supplement: Multimedia Appendix 2 [file mhealth_v12i1e50851_app2.docx]

Appendix 2: Focus-Group semi-structure guide

# Outline

- **Introduce moderator and assistant (if present) & topic/purpose of the focus group (t=0)**

*Good morning/afternoon/evening and welcome to research focus group session. Thank you for taking the time to join us to talk about use of mobile apps containing game features or rewards to motivate better medication taking. My name is Steven Tran and assisting me is __________. We're both/I am with the University of Sydney conducting this research as part of my masters. You were invited here today because we would like to hear opinions on the topic from patients taking medications such as yourself. The results will help us identify your perspectives and concerns for this use case.*

- **Outline guidelines for focus group (t=1)**
  - No right or wrong answers
  - Listen respectfully to others as they share their views, we may ask if you agree or disagree and why afterwards
  - Focus group is being recorded, one person speaking at a time
  - We would prefer you to show your camera however it we respect your decision should you choose not to use it
  - Preferred name basis always
  - Should you need to briefly leave the focus group e.g. phone call, please do so quietly and rejoin as quickly as possible.

**START RECORDING NOW**

- **Establish Rapport (t=5)**
  - *Before we start with the first question, I would like to know about each other by going around the group. Tell us your preferred name and one fun fact about yourself. I’ll start, _______ (then nominate participants).*
- **Questions:**
  - Medication adherence (define adherence) (t=10)
    - Set context: *We understand that there are many challenges with medications (e.g. AEs) but today we would like to focus specifically on taking medications as prescribed.* What would you consider possible factors that stop people from taking medications as prescribed? Do you feel these factors are applicable to your experiences? Whether positive or negative?
    - If/when you had trouble taking your medications as prescribed what helped or would help if available?
  - Mobile apps (t=20)
    - Tell us about any experiences you’ve had with using any health apps. What has been your experience?
      - Have you used a mobile app to help with medication adherence and if so, what has been your experiences with them?
      - How do you feel about mobile apps for medication adherence?
    - *RECAP their experience on features they liked.* Let’s turn our conversations to thinking about what a mobile app for medication adherence might look like?
      - What would you like it to include?

*Now that we’ve cover ……… we would like to know more about ……….*

- - Gamification (define) (t=30)
    - Have you observed the use of gamification in other aspects of your life, health or non-health related? How did you feel about it?
    - In your opinion is the use of gamification in health-related areas such as medication adherence appropriate? Why?
    - What game elements relates the most or would help with medication adherence? Why?
      - Of the suggested game elements which ones would be suitable in a mobile app?
    - What sort of game elements would you feel be counterproductive to medication adherence? Why?
  - Incentives (define) (t=45)
    - Do you use inventive/reward programs? What motivates you to use them and for how long have you used it?
    - Reflecting on the longest experience you had with an incentive program, what aspect of the incentive program made you remain adherent?
    - Has there been a time when financial incentives were not effective for you and why?
    - In your opinion is the use of financial incentives in health-related areas such as medication adherence appropriate? Why?
    - If applied in a mobile app what would you want to see?
    - What is a suitable reward or financial amount?
    - What concerns do you have about the use of financial incentives this way?
- *Thank you very much for your time and the information you shared today.* (t=59)
  - Ask if participants would be interested in getting feedback in the form of a summary after the study is complete. (collect names, irrespective of previous response on REDCap)
