# Supplementary material for: Understanding Patient Perspectives on the Use of Gamification and Incentives in mHealth Apps to Improve Medication Adherence: Qualitative Study
Source: JMIR Mhealth Uhealth. 2024 May 14;12:e50851. doi: 10.2196/50851 (PMC11134245; doi:10.2196/50851)
Supplement: Multimedia Appendix 3 [file mhealth_v12i1e50851_app3.pdf]

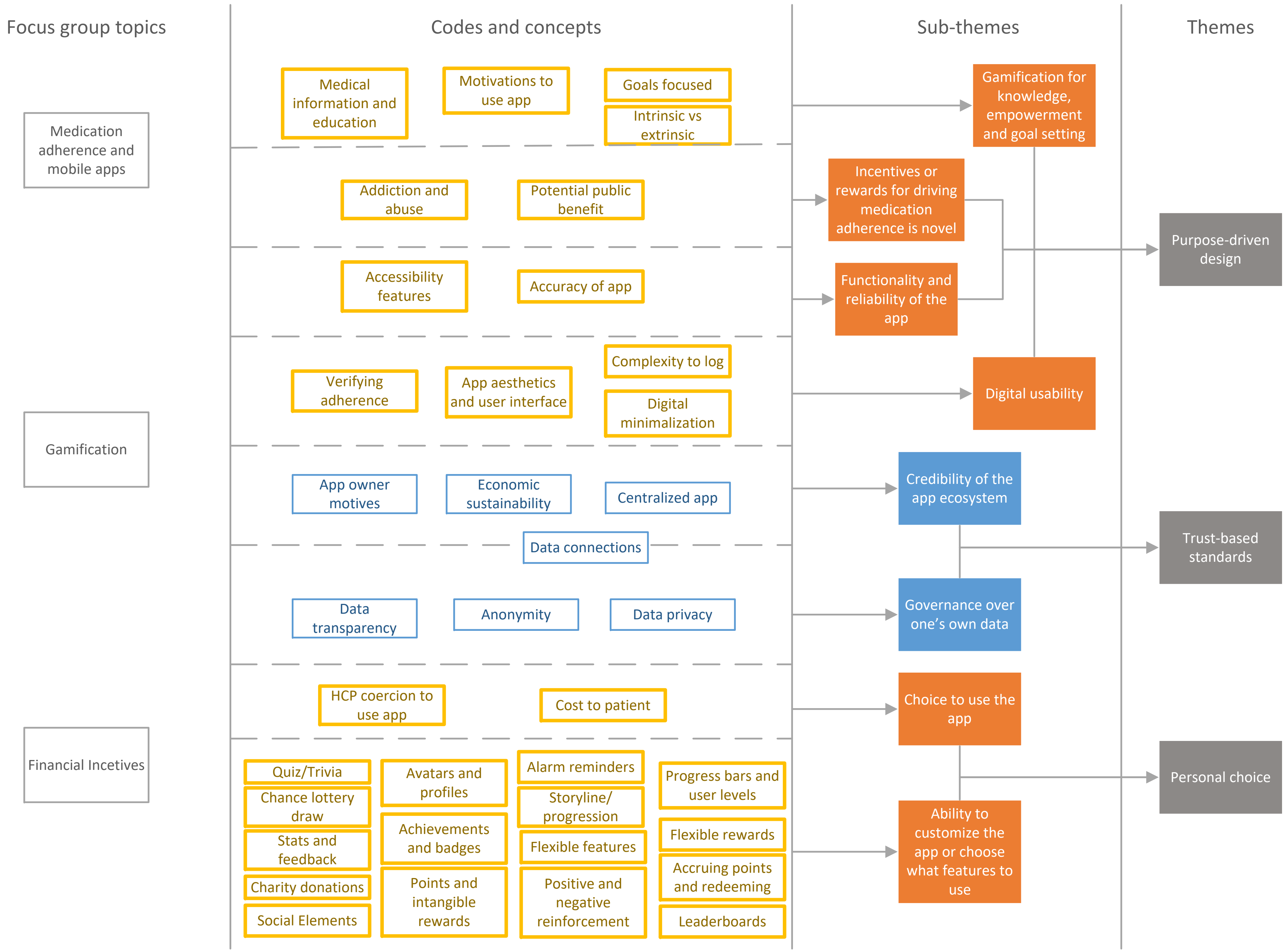

Focus group topics
Codes and concepts
Sub-themes
Themes
Medication adherence and mobile apps
Gamification
Financial Incetives
Medical information and education
Motivations to use app
Goals focused
Intrinsic vs extrinsic
Addiction and abuse
Potential public benefit
Accessibility features
Accuracy of app
Verifying adherence
App aesthetics and user interface
Complexity to log
Digital minimalization
App owner motives
Economic sustainability
Centralized app
Data connections
Data transparency
Anonymity
Data privacy
HCP coercion to use app
Cost to patient
Quiz/Trivia
Chance lottery draw
Stats and feedback
Charity donations
Social Elements
Avatars and profiles
Achievements and badges
Points and intangible rewards
Alarm reminders
Storyline/ progression
Flexible features
Positive and negative reinforcement
Progress bars and user levels
Flexible rewards
Accruing points and redeeming
Leaderboards
Gamification for knowledge, empowerment and goal setting
Incentives or rewards for driving medication adherence is novel
Functionality and reliability of the app
Digital usability
Credibility of the app ecosystem
Governance over one's own data
Choice to use the app
Ability to customize the app or choose what features to use
Purpose-driven design
Trust-based standards
Personal choice
