# Supplementary material for: Understanding Patient Perspectives on the Use of Gamification and Incentives in mHealth Apps to Improve Medication Adherence: Qualitative Study
Source: JMIR Mhealth Uhealth. 2024 May 14;12:e50851. doi: 10.2196/50851 (PMC11134245; doi:10.2196/50851)
Supplement: Multimedia Appendix 4 [file mhealth_v12i1e50851_app4.docx]

Appendix 4: Additional quotes from participants

| Sub-themes | Quotes |
| --- | --- |
| Personal health needs | - I would probably just need someone to show me the benefits and how to use it. Probably that’s the main thing for me to give an app a try. [Participant 13] - I think it's yet to be seen whether or not it's (the use of gamification in medication adherence) appropriate or not. I just don't believe it's found it's natural niche in the health industry. I think when it comes to taking medications, what is the tangible benefit that I get out of this other than, you know, improving my health? [Participant 1] - I think the reward has to not be meaningless, but just one little notch up from meaningless. I think it has to be extremely subtle. So subtle that for a patient not to worry about it but still have someone something to look forward to [Participant 5] - As long as the user fully understands and recognizes the reward and if it resonates with them, then I think they'll continue to use the app and make it actually part of that of their daily life. [Participant 5] - It would be important to figure out what the goal of medication adherence is for people, for example, before figuring out what kind of reward to give. [Participant 2] |
| Public benefit and impact | - I'm only taking one medicine at time, but I could see how an app would be really useful if you're having multiple medications because an alarm can't do that. [Participant 4] - Maybe for older people an app would help because they are more likely to be forgetful. [Participant 3] - I think people who are really tech savvy or are interested in new things would love to use an app. [Participant 19] - (The use of gamification in medication adherence) is pretty good because the long-term goal is to educate the clients and learn about the side effects of drugs and when it's safe or not safe. [Participant 15] - I think that it is appropriate (to have gamification in apps) because it's it is a good way to get people to take their medication. I guess a negative one would be I don't know if people were trying to game the system, pun intended, whether they were trying to get the points involved in it. [Participant 17] |
| Functionality and reliability | - I don't think it (game elements) would deter me from using the app if I thought the app was reliable, but I would start to feel a bit gamey. [Participant 17] - I think just talking to the app and it just records it rather than having to type. Rather than having to pick up my phone and input the medication I've taken, dosage and what time it was. [Participant 7] - I'm less likely to use the app if it’s not keeping an accurate log of things in the app. It just like it's a self-fulfilling prophecy. Once you stop using it, you don't want to keep using it. [Participant 2] - I honestly don't have much of a problem with it unless that was a slower app in general. Other than that, for me, it doesn't make any difference. [Participant 18] |
| Digital Usability | - From experience if using an app becomes like a chore and requires too much input, then it's difficult to maintain. [Participant 1] - If you take medication, you get that notification in the morning, you have to take that photo with yourself taking the medication. That's a way to track that you've taken it because you've got a photo of you physically taking it but then that could also be a bit of pressure. [Participant 8] - At an early stage in the process, it could be about the app tracking whether you get your script filled because if you don't get a script filled, then there's no way you're going to adhere to taking in medication. [Participant 9] - I'm very much into digital minimalism. If I don't have a need to install an app which is not to install the app, and that's probably the sole reason I wouldn't go around searching for an app specifically for this purpose. [Participant 2] - We are incentive driven as a race or as a people, the same as we're social creatures. I agree, those (incentivized) systems like Qantas frequent flyers and Flybuy rewards are very cryptic, and they're meant to be. I don't dispute that if it's simple and easy to use it would be enticing to me. [Participant 14] |
| Credibility of the app ecosystem | - I don't like advertising or the idea of pushing heaps of brands, but that might be a way to get the money. If the app is free, for example, that means that if you're not paying for something, then you're the product being sold. Your attention's the thing that's being sold. That means that they might need to involve companies and advertising. And I don't know if that's something that should be linked to medication, and I don't think that the government is happy to give funding. [Participant 8] - It all depends on who has created the app. So, if the app is from the government agencies I would opt yes. But if the app is from any medical research group or any pharmaceutical companies, definitely a no. [Participant 12] - If the insurance is looking at the app data or if the insurance company is providing the app. Will they use my data to size up my claims? Will it affect my ability to get insurance for me? Will it affect my ability to make claims? It just wouldn't make me comfortable. [Participant 17] |
| Governance over one’s own data | - I think clarifying where people's personal data about medication will go, whether it's linked to Medicare or linked to certain health professions. Yeah, I think just clarifying the privacy of information from the get-go is important. [Participant 2] - I could see how sharing data could be a good thing. But I think it does really depend on whether the person wants to have an app in the first place that is linked to pharmacies or their GP. [Participant 4] - When you try to fill in the forms (for financial incentives) and they ask you a lot of questions like your demographic, your age group and all. Even if they ask for my email and my name, that should be fine. But sometimes they've asked too many questions, and that won't appeal to me anymore because of data privacy concerns. [Participant 12] - I'm just in the same way that people are trying to have digital minimization. I'm trying to have data minimization. [Participant 16] - I'm probably more concerned about data security, especially in light of all the Optus and Medibank hackings. I think until there's better security around our data protection, I would feel hesitant to share data. [Participant 17] |
| Choice to use the app | - If you have to pay for the app (I would be less inclined to use the app). [Participant 19] - (I stopped using an app), because it started off as free and then changed to only certain people benefiting from the financial incentives, they had to have a card or registered with certain companies. [Participant 18] - I definitely don't think you can charge people for the app. So yeah, this is kind comes back to like most things, money. [Participant 9] |
| Ability to customize and choose what features to use in the app | - Being able to switch features on and off would be important. I think if you customize it to how you wanted to use and view the app, you're more likely to use it. So even color scheme is important. Being able to rearrange how the initial home page looks for your app, that would be pretty cool as well. [Participant 17] - If it takes a long time to set up any customization and setting up an avatar or profile, being very time poor, it would put me off. [Participant 7] - Sometimes you can customize how you want something to look and feel that if you put it in the hands of the consumer and they can customize it. It'll feel more personal, and they'll enjoy using the app more. [Participant 19] |
